# Editorial: Molecular interactions between crops and phytopathogens, Volume I: Wheat and maize

**DOI:** 10.3389/fpls.2022.979855

**Published:** 2022-07-27

**Authors:** Xiaodong Wang, Xiaojie Wang, Xiao-Ren Chen, Jianhui Wu, Jin-Ying Gou, Meixiang Zhang, Guotian Li, Lisong Ma

**Affiliations:** ^1^State Key Laboratory of North China Crop Improvement and Regulation, College of Plant Protection, Hebei Agricultural University, Baoding, China; ^2^State Key Laboratory of Crop Stress Biology for Arid Areas, College of Plant Protection, Northwest Agriculture and Forestry University, Xianyang, China; ^3^College of Horticulture and Plant Protection, Yangzhou University, Yangzhou, China; ^4^College of Agronomy, Northwest Agriculture and Forestry University, Xianyang, China; ^5^State Key Laboratory of Genetic Engineering, MOE Key Laboratory for Biodiversity Science and Ecological Engineering, MOE Engineering Research Center of Gene Technology, School of Life Sciences, Institute of Plant Biology, Fudan University, Shanghai, China; ^6^National Engineering Laboratory for Endangered Medicinal Resource Development in Northwest China, Key Laboratory of Medicinal Resources and Natural Pharmaceutical Chemistry of Ministry of Education, College of Life Sciences, Shaanxi Normal University, Xi'an, China; ^7^State Key Laboratory of Agricultural Microbiology and Provincial Key Laboratory of Plant Pathology of Hubei Province, College of Plant Science and Technology, Huazhong Agricultural University, Wuhan, China; ^8^College of Horticulture, Hebei Agricultural University, Baoding, China

**Keywords:** wheat, maize, fungal pathogen, multiple omics, hypersensitive response

Crop disease poses a significant threat to global food security as the world population continues to expand dramatically. Crops have evolved sophisticated strategies to ward off various invading phytopathogens. Accordingly, phytopathogens have evolved intricate virulent mechanisms to facilitate their infection processes. Steady progress has been achieved in understanding plant resistance to phytopathogens in the last two decades. An emerging field of studying the molecular basis of crop-pathogen interactions is gaining more attention.

Wheat and maize are the main food crops cultivated worldwide. The dominant wheat-maize rotation in many areas has remarkably impacted the cultivation environments of these two crops. In addition to traditional fungal diseases such as wheat rust, powdery mildew, and *Fusarium* head blight, soil-borne diseases, including *Fusarium* crown rot, *Gibberella* stalk rot, and sheath blight, have emerged as new threats to the global productions of wheat and maize.

This Research Topic aims to explore the application of multiple omics in understanding the molecular interactions between wheat/maize and phytopathogens; identification of novel genes or genetic loci of wheat/maize conferring resistance to phytopathogens; functional characterization of defense-related genes in wheat/maize; and discovery of pathogenicity-related factors in phytopathogens. In total, it collects nine intriguing articles from international experts in the field and covers a broad range of subjects, which allows us to divide these articles into the following two themes:

## Plant basal and induced resistance

The plant cell wall serves as the first barrier against the invasion of phytopathogens. Glucan is involved in the formation of callose, which prevents the initial infection of pathogens at the cell wall. Cheng et al. characterized a wheat glucan synthase-like gene *TaGSL22* during plant resistance to powdery mildew pathogen (*Blumeria graminis* f. sp. *tritici, Bgt*). An avirulent *Bgt* pathotype significantly upregulated the expression level of *TaGSL22*. Knocking down of *TaGSL22* by virus-induced gene silencing (VIGS) reduced callose deposition, which resulted in enhanced wheat susceptibility to *Bgt*.

Necrotrophic phytopathogens destroy basal host defense with various cell wall-degrading enzymes (CWDEs) to facilitate their infections. In contrast, host plants evolve corresponding mechanisms to counteract the activities of CWDEs. Guo et al. identified a maize gamma-aminobutyric acid transaminase (GABA-T) that directly interacted with a cellulase from corn sheath blight pathogen (*Rhizoctonia solani*). Furthermore, they demonstrated that the identified maize *GABA-T* gene and its rice homolog (*OsGABA-T*) were sufficient to suppress the cellulase-induced necrosis, while CRISPR/Cas9-mediated *OsGABA-T* knockout rice plants displayed enhanced susceptibility to *R. solani*.

As key downstream of plant defense response, various pathogenesis-related (PR) proteins function in the apoplastic space during the onset of plant-pathogen interaction. Zhao J. et al. revealed that a wheat lipid transfer protein TaLTP3, also known as a homolog of PR14, was positively correlated with plant resistance to leaf rust. Overexpression of the *TaLTP3* gene in the transgenic wheat lines resulted in enhanced rust resistance and higher expression of *TaPR1a* in response to the infection of the model bacterial pathogen *Pseudomonas syringae* pv. *tomato* DC3000. Further investigation indicated that TaLTP3 directly interacted with TaPR1a in the apoplastic space to co-regulate the plant defense response.

Key regulators and components in early responses of plant resistance to phytopathogens may have great potential in interpreting the mechanism of plant defense responses. Using multiple omics techniques, Tang et al. profiled the regulatory network of a maize *Gibberella* stalk rot (GSR)-resistant gene *ZmCCT* at the early infection stage. The *ZmCCT*-mediated maize resistance to *Gibberella* stalk rot caused by *Fusarium graminearum* were associated with activations of pattern-triggered immunity (PTI), phytohormone pathways of salicylic acid (SA) and auxin, and accumulations of phenylalanine metabolites.

Systemic acquired resistance (SAR) was another critical add-on for plant defense response beyond the hypersensitive response (HR). The action of some biocontrol agents was intensively associated with SAR. Boamah et al. reported that a biocontrol fungus *Trichoderma longibrachiatum* (TG1) with positive effects on wheat tolerance to salt stress and resistance to *Fusarium* crown rot caused by *F. pseudograminearum*. Moreover, expression levels of *PR* genes were significantly increased in response to TG1.

## Hypersensitive response

Activation of plant major resistance (*R*) genes in response to biotrophic phytopathogens often leads to hypersensitive responses (HR) at the infection site following the gene-for-gene theory. Many *R* genes derived from crop wild relatives are widely used in breeding practice. Jin et al. identified a stripe rust resistance gene *YrM8664-3* in a wheat-*Leymus mollis* introgression line. *YrM8664-3*, located on wheat chromosome 4AL, encoded a protein with plastid lipid-associated proteins (PAP)_fibrillin domain (TaFBN). *TaFBN* conferred high resistance to stripe rust in transgenic wheat lines.

Myeloblastosis (MYB) transcription factors are vital components in plant development and resistance to various stresses. Zhu et al. cloned a member of the R2R3-MYB superfamily in wheat and designated it as *TaMYB29*. Transient expression of *TaMYB29* in tobacco leaves triggered pathogen-independent cell death associated with increased accumulation of reactive oxygen species (ROS). Conversely, silencing of *TaMYB29* by VIGS significantly reduced the wheat resistance to stripe rust with reduced ROS accumulation.

Specific mutations on plant *R* genes that exhibited autoactivated HR are considered as valuable genetic resources to explore the mechanism of cell death in the plant. Ge et al. revealed the transcriptomic and metabolomic profiles of a maize auto-active HR mutant *Rp1-D21*. Further investigation indicated that two maize UDP-dependent glycosyltransferase (*ZmUGTs*) genes partially suppressed the HR triggered by Rp1-D21 in a protein interaction-independent manner.

Interestingly, HR-like black necrotic lesions (HR-BNL) often occur around the leaf infection sites of *Fusarium* head blight (FHB) in resistant wheat cultivars. Zhao L. et al. investigated the HR-BNL response in the leaf samples of a resistant wheat cultivar Sumai 3 inoculated with four disinct *F. graminearum* isolates. Flavonoid metabolites were identified and proved to be involved in the formation of HR-BNL. SA signaling pathway associated with ROS burst positively regulated FHB resistance in wheat with HR-BNL.

Overall, this Research Topic presents a broad range of articles that describe the application of multi-omics approaches in different wheat/maize-fungus pathosystems and expand our knowledge toward understanding the molecular interactions between wheat/maize and phytopathogens. Notably, several collected articles focusing on the role of HR in crop resistance have provided new insights into the molecular mechanism of crop major resistance genes.

## Author contributions

All authors listed have made a substantial, direct, and intellectual contribution to the work and approved it for publication.

## Funding

XiaodW was supported by the Provincial Natural Science Foundation of Hebei (C2022204010 and C2021204008) and State Key Laboratory of North China Crop Improvement and Regulation (NCCIR2021ZZ-4). XiaojW was supported by the Shaanxi Innovation Team Project (2018TD-004). X-RC was supported by the National Natural Science Foundation of China (31871907 and 31671971) and Jiangsu Agriculture Science and Technology Innovation Fund (JASTIF) [CX(20)3125]. JW was supported by the Key R&D Program of Shaanxi Province in China (2021ZDLNY0-01). J-YG was supported by the National Natural Science Foundation of China (31972350). MZ was supported by the National Natural Science Foundation of China (32072399 and 31672008) and the Fundamental Research Funds for the Central Universities (GK202201017). GL was supported by the National Natural Science Foundation of China (32172373). LM was supported by the Hundred Talents Program for the introduction of high-level overseas talents in Hebei Province (E2020100004).

## Conflict of interest

The authors declare that the research was conducted in the absence of any commercial or financial relationships that could be construed as a potential conflict of interest.

## Publisher's note

All claims expressed in this article are solely those of the authors and do not necessarily represent those of their affiliated organizations, or those of the publisher, the editors and the reviewers. Any product that may be evaluated in this article, or claim that may be made by its manufacturer, is not guaranteed or endorsed by the publisher.

